# Dual processing theory and expertsʼ reasoning: exploring thinking on national multiple-choice questions

**DOI:** 10.1007/s40037-015-0196-6

**Published:** 2015-08-05

**Authors:** Steven J. Durning, Ting Dong, Anthony R. Artino, Cees van der Vleuten, Eric Holmboe, Lambert Schuwirth

**Affiliations:** 1 Uniformed Services University of the Health Sciences, 4301 Jones Bridge Road, 20814 Bethesda, MD USA; 2 Maastricht University, Maastricht, The Netherlands; 3 Accreditation Council for Graduate Medical Education, 515 North State Street, 60654 Chicago, IL USA; 4 Flinders University, Adelaide, Australia

**Keywords:** clinical reasoning, assessment, dual-process theory

## Abstract

**Background:**

An ongoing debate exists in the medical education literature regarding the potential benefits of pattern recognition (non-analytic reasoning), actively comparing and contrasting diagnostic options (analytic reasoning) or using a combination approach. Studies have not, however, explicitly explored faculty’s thought processes while tackling clinical problems through the lens of dual process theory to inform this debate. Further, these thought processes have not been studied in relation to the difficulty of the task or other potential mediating influences such as personal factors and fatigue, which could also be influenced by personal factors such as sleep deprivation. We therefore sought to determine which reasoning process(es) were used with answering clinically oriented multiple-choice questions (MCQs) and if these processes differed based on the dual process theory characteristics: accuracy, reading time and answering time as well as psychometrically determined item difficulty and sleep deprivation.

**Methods:**

We performed a think-aloud procedure to explore faculty’s thought processes while taking these MCQs, coding think-aloud data based on reasoning process (analytic, nonanalytic, guessing or combination of processes) as well as word count, number of stated concepts, reading time, answering time, and accuracy. We also included questions regarding amount of work in the recent past. We then conducted statistical analyses to examine the associations between these measures such as correlations between frequencies of reasoning processes and item accuracy and difficulty. We also observed the total frequencies of different reasoning processes in the situations of getting answers correctly and incorrectly.

**Results:**

Regardless of whether the questions were classified as ‘hard’ or ‘easy’, non-analytical reasoning led to the correct answer more often than to an incorrect answer. Significant correlations were found between self-reported recent number of hours worked with think-aloud word count and number of concepts used in the reasoning but not item accuracy. When all MCQs were included, 19 % of the variance of correctness could be explained by the frequency of expression of these three think-aloud processes (analytic, nonanalytic, or combined).

**Discussion:**

We found evidence to support the notion that the difficulty of an item in a test is not a systematic feature of the item itself but is always a result of the interaction between the item and the candidate. Use of analytic reasoning did not appear to improve accuracy. Our data suggest that individuals do not apply either System 1 or System 2 but instead fall along a continuum with some individuals falling at one end of the spectrum.

## Introduction

A physician’s practice is centred around clinical reasoning—arriving at the correct diagnosis and a treatment strategy that is specific to a patient’s circumstances and preferences. Assessing clinical reasoning, however, is difficult given the inability to directly observe a physician’s thought processes and the resulting necessity to always infer their reasoning from observable behaviour [1].

Developments in the assessment of clinical reasoning have come a long way since the reliance on more or less authentic patient simulations in the 1960s–1980s [2, 3]. Back then, the prevailing notion was that presenting candidates with a clinical problem, asking them to request all the relevant information and then scoring all diagnostic and therapeutic decisions was the optimal way to assess clinical reasoning. This idea, however, proved to be incorrect, especially for high-stakes decisions, such as licensure, where these approaches were shown to be seriously flawed both in their reliability and construct validity [4].

Since then, clinical reasoning developments have moved in two main directions: one focusing on the intermediate or final outcomes of the decision-making process, such as key-feature approach testing and extended-matching items [5, 6], and others focusing on the underlying process itself, such as the script concordance test [7]. The assumption of the former is that a correct outcome of the process reflects a correct process, while the latter approach asserts that a correct process would lead to a correct outcome. We have learned from all these developments, though, and it now appears that the matter of clinical reasoning and its assessment are far more complicated than was assumed in those early decades [1].

In the general context of naturalistic decision making, theories of metal models being defined as metal representations argued that metal models are an interplay of knowledge, perception, and comprehension. Further, metal models can be analyzed in terms of concepts and their relations. A leading theory used to understand the cognitive processes of clinical reasoning is dual-process theory [8], which distinguishes two processes: non-analytic and analytic reasoning. The former, also called System 1, relates to fast and effortless unconscious thinking (e.g. pattern recognition). The latter, also called System 2, denotes the slow and effortful process of problem solving by conscious analysis. This theory has been applied in many other fields in addition to clinical reasoning [9].

The discourse about both systems in clinical reasoning is complex. Generally, with respect to System 2, it is assumed that when it comes to reasoning, the clinician actively compares and contrasts features of the problem at hand with features of prototypical cases or abstract representations in his/her memory to find the optimal solution [8]. Immediate and more intuitive solutions—as an effect of System 1 processes—are seen as the result of retrieving the most appropriate exemplar or script for the problem at hand from memory. Better System 2 processing is associated with higher general ability, including intelligence. Superior System 1 processing is seen as the result of extensive experience and expertise [8]. This is plausibly related to the findings in expertise research and the notion of deliberate practice, which basically agree that becoming an expert is a matter of building a large database of robust prototypes and well-retrievable experiences or an array of flexibly applicable problem-solving strategies [10].

Research shows that the best predictor for successful (diagnostic) clinical reasoning is the quality of System 1 processing—in particular the probability of the correct diagnosis being considered by the clinician [11]. Research also suggests that the most common source of diagnostic error is the failure to engage in System 2 reasoning when System 1 is not sufficient [11]. There are, however, still unresolved issues in the relation between both systems and successful clinical reasoning. For example, one perhaps unexpected finding is that non-analytical reasoning is more effective if the number of features that need to be considered in the problem solving process is large, and that the analytical System 2 route is more effective in cases where the problem is ‘simpler’ [12, 13]. Additionally, others argue that the optimal clinical reasoning strategy/ies is likely dependent upon the situation or, better, the specific relation between the complexity of the problem and the level of expertise of the clinician [14, 15].

This creates a paradox, however, as one expert may see a problem as simple that other experts may see as difficult. Following Dijksterhuis’ argument it would then be best for the most expert physician to engage in analytic reasoning because his/her expertise allows him/her to reduce the problem to a few information-rich chunks (due to non-analytic reasoning) which can be easily analyzed to produce the best solution. For a novice, on the other hand, this would not be possible and he/she would be best served by approaching the problem non-analytically. But this is paradoxical as the novice has not had the time or experience to develop sufficient exemplars or chunks to rely on non-analytical reasoning.

This raises the question whether our conceptions of difficulty or complexity of a test item for the assessment of clinical reasoning are in line with theories on clinical reasoning. Often, aggregate performance data are used to distinguish hard from easy problems. Dual processing theory and those arguing that reasoning is situation specific would rather support an individualized notion, namely that an item can only be hard or easy for a person depending on his/her specific experience with the (type of) problem at hand [16, 17]

In other words, the contention is that hard versus easy or complex versus straightforward is mainly determined by the interaction between the test taker and the problem, rather than being predominantly a feature of either, which differs from our current psychometric stance. But even if this were the case, the question remains whether this interaction between the problem and the candidate can be seen as a relatively stable facet or if it can be influenced by other factors. Fatigue and sleep deprivation, for example, have been shown to be associated with medical errors and thus a variety of other situational influences may impact on the optimal communication between both process strategies in a dual-process architecture [18]. Thus, fatigue could alter the relationship between candidate and assessment item. Finally, current assumptions view non-analytic and analytic reasoning more as a dichotomous system (e.g. you are using one or the other system vs. a more continuous use of both)—this stance should be explicitly tested.

In this study involving board-certified internal medicine physicians, we sought to understand the relationship between faculty physicians’ (experts) reasoning processes, assessment items used for national exam purposes as problems, and the psychometrically determined difficulty. In addition, we sought to study the influence sleep deprivation, as one situation specific factor, would have on this relationship.

## Method

Board-certified internists answered a series of multiple choice items that had been used in a large sample of participants with known psychometric characteristics followed by a formal think-aloud procedure so that their answers could be linked with their thought processes.

### Participants

Following informed consent, 22 board-certified internal medicine attending physicians (faculty or experts) with faculty appointments at the Uniformed Services University (USU) participated in the study. The study was approved by the Institutional Review Boards of the USU and Walter Reed Army Medical Center.

### Measurements

#### Multiple-choice questions (MCQs)

We used validated MCQs from the American Board of Internal Medicine (ABIM) and National Board of Medical Examiners (NBME) in this investigation. These organizations are responsible for licensing physicians in the US and they validate the appropriateness of their items by subjecting them to a rigorous internal content review and performance analysis.

The vignette-based MCQs selected required the integration and synthesis of data, and items queried ‘What is the most likely diagnosis?’ (i.e. the examination items were designed to assess clinical reasoning at least partly as a System 2 process [19]. Participants answered a total of 32 questions: 16 NBME items (United States Medical Licensing Examination [USMLE] Step 2 Clinical Knowledge items) and 16 ABIM items (Maintenance of Certification [MOC] MCQ). We selected questions that fit on a single screen and contained only words (i.e. there were no chest X-rays or other images) and with good item discrimination indices. Participants pushed handheld buttons for answer options ‘A’ to ‘E’. Item accuracy was used as a dependent variable.

As these were items used in national examination we could select them based on whether they were considered hard or easy items. We intentionally selected easy and hard items from each of the two question sources (total of 14 easy and 18 hard items; 16 of these items were cardiology and 16 were rheumatology) as we sought to explore the interaction of reasoning performance with item difficulty, a dependent variable. Easy and hard items were defined by the percentage of test takers answering the items correctly; easy items were defined as the ones with a *p*-value higher than .70 and hard items with a *p*-value of lower than .20.

Participants were instructed to push the button as soon as they were done reading the item, which ended with a diagnostic question, per above, but without A–E options. Participants had up to 60 s to do so. Next, participants entered the answering phase where they had up to 7 s to select the A–E answer. Time was recorded for pushing the buttons to the 10^− 8 s^ level.

#### Think-aloud

Immediately following completion of the items, participants underwent a formal think-aloud procedure so that we could explore thought processes, through the lens of dual process theory, with answers. These think-aloud data were used as an independent variable. Think-aloud protocols are a way to explore the thought processes of a participant while they complete a task [20, 21]. Currently, think-aloud protocol methodology, either during the task or retrospectively after the task, is seen as an acceptable procedure to capture thought processes such as clinical reasoning [10, 22]. We chose to conduct the think-aloud activity retrospectively as we did not want it to interfere with actual answering and, in particular, with non-analytic reasoning. The think-aloud procedure was used to differentiate examinee use of System 1, System 2, a combination of both, or guessing, on an item-by-item basis. As we sought to use dual-process theory as our theoretical lens, we also counted the number of words uttered on the think-aloud with each MCQ, as well as number of concepts to further help with distinguishing System 1 and System 2 use.

#### Additional measures

As a measure of fatigue (an independent variable) prior to answering questions, participants completed a survey, containing questions about hours worked in the days just prior to completing this study. While answering items, we captured reading time and answering time as well as if the item was answered correctly or not. Reading and answering time were also used to inform the use of dual processing strategy (nonanalytic expected to be associated with shorter reading and answering time as it entails pattern recognition) and accuracy of answering items was important to capture so that we could explore differences in strategies with answering items correctly or incorrectly.

### Analysis

This was a study that employed numeric survey data, timing data from reading and answering items, and qualitatively analyzed think-aloud data. Think-aloud data were audiotaped and transcribed. Two coders subsequently scored each item for strategy use (analytic, nonanalytic, guess, or combination therein) and number of concepts. These comments were coded independent of data on item difficulty.

The quantitative analyses consisted of four parts. First, we calculated each participant’s average think-aloud word count, number of concepts, reading time, and answering time across MCQs, and the total number of correct answers. Word count, number of concepts and times were used to help distinguish analytic from non-analytic reasoning in conjunction with our think-aloud coding procedure. Next, we performed descriptive statistics of these measures and conducted Pearson correlation analysis between these measures as well as working hours in the last 3 days and last 48 hours reported by the participants in the pre-survey to address the question of the influence of fatigue on reasoning process use. We repeated this procedure for all MCQs, hard MCQs only, and easy MCQs only; the last two procedures were used to inform the investigation of the impact of hard or easy categories on dual process use and accuracy.

We then used the individual question as the unit of analysis and provided an overview of the frequencies of think-aloud processes in six situations—getting the hard questions right, getting the hard questions wrong, getting the easy questions right, getting the easy questions wrong, total questions right, and total questions wrong. We calculated each participant’s frequency of expressing non-analytic reasoning, combined approach, analytic reasoning, and guessing in think-aloud. We investigated the Pearson correlations between these categorical measures (reasoning by think-aloud coding), item accuracy and hours worked in the last 3 days and last 48 hours. Again, we did this procedure for all MCQs, hard MCQs only, and easy MCQs only. In addition, we performed t-tests to see whether the participants’ expression of these think-aloud processes differed between hard and easy questions. Finally, we performed multiple regression analysis to examine the influence of expressing combined approach, analytic reasoning, and non-analytic reasoning, considered together, on number of MCQs answered correctly.

## Results

Table 1 displays item difficulty by national standards, reasoning strategy by think-aloud categorization and percentage correct. The following codes were used following review of the data and discussions leading to consensus following coding of approximately 15 % of the data. Each MCQ think-aloud was given one code. The codes were guessing, analytic, non-analytic, combined, and other (the last referring to utterances that could not be coded). Guessing involved explicitly stating that one was unsure about the correct answer.

**Table 1:** Percentages correct and incorrect answers for the hard items and easy items (by *p*-value) and over the total set of items by type of reasoning

	Non analytical	Combined	Analytical	Guessing	Rest
‘Hard’ correct	48	34	10	3	5
‘Hard’ incorrect	30	29	18	16	6
‘Easy’ correct	61	28	6	4	1
‘Easy’ incorrect	39	28	11	19	3
Total correct	56	30	7	4	3
Total incorrect	33	29	16	17	5

*Examples: I have no idea.*

*My answer is a complete guess.*

Analytic reasoning involved explicit comparing and contrasting diagnoses (or other key data) by the examinee.

*Examples: Based on the data provided in this question, the answer is either X or Y which is based on how one weighs the supporting data, which include the following….*

*The answer is either B or C and I am leaning towards B because of the following features….*

Nonanalytic reasoning was recognized when the examinee explicitly demonstrated that they were chunking data, forming a pattern.

*Examples: The patient has X, Y, and Z—this is the diagnosis.*

*So, it is clear that this patient has heart failure.*

Combined strategy was used when the participant vocalized using both nonanalytic and analytic reasoning.

*Example: These symptoms and findings mean that the patient has X diagnosis, but this additional finding suggests diagnosis Y or X.*

Regardless of whether the questions were classified as ‘hard’ or ‘easy’ depending on their national item statistics or were analyzed as a total group, non-analytical reasoning led to the correct answer more often than to an incorrect answer. In fact, in all those item groups the chance of a correct answer was around 60 %. When a combined approach was used this chance dropped to only 50 % and with analytical reasoning it dropped to 30–35 %. As the literature has found that the ability to correctly solve a problem by non-analytical reasoning is dependent on exposure to this or similar problems [17], the table seems to suggest that the difficulty of an item may be more strongly related to the individual experience of the candidate than to an innate property of the item, and a *p*-value is more an indication of the probably that a candidate has been exposed to the problem at hand than of the complexity of the problem.

Table 2 shows the correlation between the variables used in the study.

**Table 2:** Descriptive statistics of the measures and Pearson correlations between them when all MCQs, only hard MCQs, or only easy MCQs were included

Measures	Mean	SD	1	2	3	4	5	6	7
1. Average word count	126.80	40.66	–	.64^a^	− 0.09	− 0.34	0.24	0.49^b^	0.57^a^
	**136.35**	**45.53**	**–**	**0.68** ^**a**^	**− 0.24**	**− 0.28**	**0.33**	**0.47** ^**b**^	**0.54** ^**b**^
	*114.53*	*35.54*	*–*	*0.56* ^*a*^	− *0.53* ^*b*^	− *0.27*	*0.07*	*0.53* ^*b*^	*0.60* ^*a*^
2. Average number of concepts	6.67	2.35		–	0.12	− 0.35	0.17	0.40	0.54^b^
	**7.25**	**2.48**		**–**	**− 0.18**	**− 0.24**	**0.24**	**0.42**	**0.55** ^**b**^
	*5.91*	*2.26*		*–*	*− 0.44*	− *0.40*	*0.13*	*0.35*	*0.50* ^*b*^
3. Average reading time	26372.50	7839.84			–	0.06	0.20	0.08	0.18
	**27381.91**	**6733.25**			–	**− 0.002**	**0.19**	**− 0.05**	**− 0.13**
	*25508.05*	*5901.04*			*–*	*0.39*	*0.07*	*− 0.08*	*− 0.18*
4. Average answering time	6526.87	667.00				–	− 0.64^a^	0.10	0.06
	**7049.51**	**756.03**				–	**− 0.35**	**− 0.09**	**− 0.06**
	*5854.90*	*816.77*				*–*	*− 0.63* ^*a*^	*0.30*	*0.18*
5. Total number of correct answers	13.55	3.25					–	− 0.14	− 0.11
	**5.40**	**2.14**					–	**− 0.12**	**− 0.07**
	*8.15*	*1.79*					*–*	*− 0.11*	*− 0.12*
6. Hours worked in the last 3 days	25.10	10.50						–	0.95^a^
7. Hours worked in the last 48 hours	17.23	6.83							–

^a^ *P* < 0.01.

^b^ *P* < 0.05.

The number in the first line of each cell was the statistic when all MCQs were included. In the second line the number in bold was the statistic when only hard MCQs were included and the number in the third line in italic was the statistic when only easy questions were included.

Significant correlations were found between the numbers of hours worked with word count and numbers of concepts used in the reasoning. This would support our assumption that fatigue has an influence on a person’s ability to use non-analytical reasoning process when solving a problem. There was a significant negative relationship between answering time and the probability of a correct answer (− 0.64). Notably, however, there was no significant correlation found between the hours worked and the total number of correct answers.

The correlational pattern in Table 3 is less clear. Although a significant negative correlation was found between one measure of fatigue (number of hours worked in the last 3 days) and non-analytical reasoning, this was not the case with the other measure of fatigue (number of hours worked in the last 48 h). Again, no direct relationship between fatigue and correctness of the answers could be demonstrated.

**Table 3:** Pearson correlations between analytic, combined, and nonanalytic think-aloud processes with correctness and working hours when all MCQs, only hard MCQs, or only easy MCQs were included

Measures	1	2	3	4	5	6	7
1. Non-analytic reasoning	–	− .071^a^	− 0.35	0.14	− 0.24	− 0.48^b^	− 0.44
	–	**0.64** ^**a**^	**− 0.32**	**0.01**	**− 0.27**	**− 0.32**	**− 0.32**
	*–*	*0.74* ^*a*^	*− 0.30*	*− 0.02*	*− 0.26*	*− 0.57* ^*a*^	*− 0.50* ^*b*^
2. Combined approach		–	− 0.24	− 0.52^b^	0.43	0.41	0.35
		–	**− 0.22**	**− 0.52** ^**b**^	**0.22**	**0.44**	**0.43**
		*–*	− *0.37*	− *0.30*	*0.51* ^*b*^	*0.29*	*0.19*
3. Analytic reasoning			–	0.02	− 0.15	0.20	0.25
			–	− **0.05**	**0.28**	**0.01**	**0.08**
			*–*	*0.14*	− *0.39*	*0.43*	*0.44*
4. Guessing				–	− 0.27	− 0.41	− 0.45^b^
				–	**− 0.24**	**− 0.41**	**− 0.48** ^**b**^
				*–*	*0.03*	− *0.18*	− *0.09*
5. Correctness					–	− 0.14	− 0.11
					–	**− 0.12**	**− 0.07**
					*–*	− *0.11*	− *0.12*
6. Hours worked in the last 3 days						–	0.95^a^
7. Hours worked in the last 48 h							–

^a^ *P* < 0.01.

^b^ *P* < 0.05.

The number in the first line of each cell was the statistic when all MCQs were included. In the second line the number in bold was the statistic when only hard MCQs were included and the number in the third line in italic was the statistic when only easy questions were included.

Finally, Table 4 presents the results of multiple linear regression models when the think-aloud processes of combined approach, analytic reasoning, and non-analytic reasoning were all entered as explanatory variables for the outcome of number of correct answers. When all MCQs were included, 19 % of the variance of correctness could be explained by the frequency of expression of these three think-aloud processes. If only easy MCQs were taken into account, this variance increased to 32 %. If only hard MCQs were included, this variance was 16 %.

**Table 4:** Multiple linear regression models to examine the associations between think-aloud processes and correctness of answers when all MCQs, only hard MCQs, or only easy MCQs were included

Explanatory variables	Unstandardized coefficient	Standard error	Standardized coefficient	Total model *R* ^*2*^	*F*-value
Combined approach	0.34	0.29	0.56		
	**0.23**	**0.24**	**0.35**		
	*0.14*	*0.50*	*0.21*		
Analytic reasoning	0.04	0.40	0.04		
	**0.38**	**0.30**	**0.38**		
	*− 0.53*	*0.72*	*− 0.38*		
Non-analytic reasoning	0.11	0.32	0.17	0.19	1.25
	**0.05**	**0.25**	**0.07**	**0.16**	**1.05**
	*− 0.15*	*0.50*	*− 0.22*	*0.32*	*2.49*

The number in the first line of each cell was the statistic when all MCQs were included. In the second line the number in bold was the statistic when only hard MCQs were included and the number in the third line in italic was the statistic when only easy questions were included.

## Discussion

In this study we sought to better understand the relationship between faculty physicians’ (experts) reasoning processes and psychometrically determined difficulty of national examination assessment items. In addition, we studied the influence that sleep deprivation might have on this relationship. Further, we tried to contribute to the ongoing debate that exists in the medical education literature regarding the potential benefits of analytic, nonanalytic, and combined processes with limited data exploring verbalized thought processes.

We believe the results are noteworthy with respect to expert performance, despite the small-scale nature of the study. First, we found evidence underpinning the notion that difficulty of an item in a test is not a systematic feature of the item itself but always a result of the interaction between the item and the candidate. This is notable because generally the interaction between candidate and item (PxI) is treated as part of the error term [23]. *P*-values—as in classical test theory—are therefore more an indication of the probability that a candidate has had exposure to the problem described in the item than the intrinsic complexity of the item itself. This is not completely counter-intuitive: for example reading is quite a complex ability, yet it is something we typically do without much analytical thinking.

Another finding from our cohort of experts is the apparent artificiality of the dichotomy between System 1 and System 2. Often it is suggested that humans solve problems by either using one system or the other. We think, and our results support, that this is at least debatable. The use of both processes simultaneously (System 1 and System 2) is also consistent with work from other fields [9]. The cases in which the answer was provided in one single concept may be indicative of pure System 1 processing, but in all other cases the concepts mentioned and manipulated in the short-term memory were chunks of information and as such again recognized patterns. To illustrate what we mean we will use the reading analogy further. When reading, recognizing letters is a non-analytical process for the more novice reader, recognizing (simple) words for the intermediate and recognizing complicated, long words and perhaps whole sentences of the expert reader. All apply both analytical and non-analytical processes but the chunks they process are more or less information rich. Our data suggest that it is not either System 1 or System 2 that the subjects applied but anywhere on the scale and some more at the extremes. In this view, the non-analytical extreme would be processing the whole problem as one single chunk.

In the introduction we described a paradox that easy problems are best solved by analytical reasoning and multi-factorial complex problems best by non-analytical reasoning, and yet for the expert complex problems are simple and for the novice simple problems can be complex. Our results suggest that this indeed is a paradox and not a contradiction. If the difficulty of a problem is mainly the result of the interaction between the candidate and the problem at hand, it means that a problem that can be solved with large chunks of non-analytical reasoning is not a difficult problem (it might be to others) and therefore analytical reasoning to manipulate large chunks in their working memory would require little effort. For a novice, on the other hand, analytically reasoning through a problem that for them is still multi-factorial (because it has to be processed in many small chunks) surpasses the limitation of their cognitive architecture [24]. This would underpin further the notion in expertise theory that one can only be an expert at a certain problem by (repeated) exposure and learning [17, 22] and that someone who is regarded as an expert is only seen as such because he/she is an expert at the many individual problems he/she has had the time and exposure to master. So being an expert is not only a matter of quality but rather of quantity. In summary, our findings do not contradict but rather support and offer a further explanation for context specificity and idiosyncrasy of problem solving.

An additional notable finding is the relationship between fatigue and numbers of concepts and words in the reasoning process. If fatigue were to lead to more diagnostic errors due to premature closure, one would have expected an inverse relationship. Our correlation between number of words and concepts in the reasoning, however, suggests that fatigue impacts on diagnostic accuracy because it induces an impairment of the non-analytical process and therefore requires more analytical reasoning for a problem that the person would be able to solve non-analytically had he/she not been fatigued or sleep deprived. In other words, instead of leading to premature closure one could suggest it leads to ‘premature opening’. We must reiterate, however, that the numbers of our study are small and that the correlational pattern is not equivocal. The validity of using the hours worked in the last 3 days and 48 h as a measure of fatigue is yet to be investigated more closely with a larger sample. Yet there were no significant correlations contradicting our conclusions.

The limitations of our study include the relatively small sample size. One could also argue whether written questions are the best proxy for actual practical clinical reasoning. Unfortunately, using a think-aloud protocol in real time in actual practice is not possible. The items, on the other hand, were taken from a well-validated national test and had all undergone rigorous quality assurance. We believe our findings warrant further research and perhaps replication of this study on a larger scale to obtain clearer correlation patterns.

### Essentials:

- We found evidence underpinning the notion that difficulty of an item in a test is not a systematic feature of the item itself but always a result of the interaction between the item and the candidate.
- Another finding from our cohort of experts is the apparent artificiality of the dichotomy between System 1 and System 2.
- Fatigue impacts on diagnostic accuracy because it induces an impairment of the non-analytical process and therefore requires more analytical reasoning for a problem that the person would be able to solve non-analytically had he/she not been fatigued or sleep deprived.
